# Cellular absorption of small molecules: free energy landscapes of melatonin binding at phospholipid membranes

**DOI:** 10.1038/s41598-020-65753-z

**Published:** 2020-06-08

**Authors:** Huixia Lu, Jordi Marti

**Affiliations:** Department of Physics, Technical University of Catalonia-Barcelona Tech, B4-B5 UPC Northern Campus, Barcelona, Catalonia Spain

**Keywords:** Biological physics, Membrane trafficking, Computational biophysics

## Abstract

Free energy calculations are essential to unveil mechanisms at the atomic scale such as binding of small solutes and their translocation across cell membranes, eventually producing cellular absorption. Melatonin regulates biological rhythms and is directly related to carcinogenesis and neurodegenerative disorders. Free energy landscapes obtained from well-tempered metadynamics simulations precisely describe the characteristics of melatonin binding to specific sites in the membrane and reveal the role of cholesterol in free energy barrier crossing. A specific molecular torsional angle and the distance between melatonin and the center of the membrane along the normal to the membrane *Z*-axis have been considered as suitable reaction coordinates. Free energy barriers between two particular orientations of the molecular structure (folded and extended) have been found to be of about 18 kJ/mol for *z*-distances of about 1–2 nm. The ability of cholesterol to expel melatonin out of the internal regions of the membrane towards the interface and the external solvent is explained from a free energy perspective. The calculations reported here offer detailed free energy landscapes of melatonin embedded in model cell membranes and reveal microscopic information on its transition between free energy minima, including the location of relevant transition states, and provide clues on the role of cholesterol in the cellular absorption of small molecules.

## Introduction

The main components of human cell membranes are phospholipids, cholesterol and different sorts of proteins, all inside a salty aqueous solution. Whereas phospholipids form the essential backbone of a biomembrane, cholesterol (*C*_27_*H*_46_O) can modulate some of its most important structural and mechanical properties, in particular its fluidity, in such a way that concentration of cholesterol can tune-up a phase transition from liquid disordered to liquid ordered states^1,2^. In this paper we have focused our efforts in the study of zwitterionic phospholipid membranes that can help understand basic biological membrane functions and their interaction with specific small molecules. As an example of a prototype membrane, we considered the one formed by dimyristoylphosphatidylcholine (DMPC, *C*_36_*H*_72_*NO*_8_*P*) lipids, a phospholipid species employed in many experimental works, since its gel to liquid phase transition happens at a relatively low temperature (around 295.5 K)^3^. A large number of simulations have already been published on DMPC membranes^4–11^, often considering the influence of cholesterol in aqueous ionic environments^2,12–18^ as well as numerous reports based on experimental techniques (neutron scattering, X-ray, infrared reflection absorption spectroscopy, NMR, etc.) have been published throughout the last decades (see for instance refs. ^19–25^).

Besides the composition of the membrane, the role of proteins and drugs and their interactions with cell membrane structures is undoubtedly a relevant field of research. In this work we have considered the introduction into the lipid bilayer structure of a small biological probe: the hormone melatonin (N-acetyl-5-methoxytryptamine, MEL, *C*_13_*H*_16_*N*_2_*O*_2_)^26^. Melatonin is a neurohormone including an indole derivative with an attached flexible peptide-like side chain secreted at the pineal gland^27^ and it is derived from tryptophan, through conversion into serotonin^28^. It has been observed that melatonin is able to cross most physiological barriers, such as the blood-brain barrier^29,30^. So, melatonin may help to control brain function^31^, and it has also interesting immunotherapeutic potential in both viral and bacterial infections^32^, it is able to help humans to the regulation of biological rhythms, to induce sleep, to work as a strong antioxidant and it also contributes to the protection of the organism from carcinogenesis and neurodegenerative disorders such as in Alzheimer’s disease^33,34^. A number of works have been focused on the structure and interactions of melatonin with zwitterionic membranes^35,36^, and several experiments and simulations suggest that small solutes such as tryptophan and melatonin are bound to the phosphate and carbonyl regions of phospholipid species^26,37–39^. Other studies based on the binding of melatonin at ion channels in lymphocytes indicated an active role of melatonin in reversible blocking of the channel and its possible activity as an immunological agent^40^. While bound to membranes in aqueous solution, strong hydrogen-bonds between the small molecule and the phospholipids belonging to the membrane have been located in a variety of possible configurations^41^. Although the binding constant and Gibbs free energy of a probe adsorption can be estimated from experiments^26,39^, detailed studies of the relative stability of different bound states are still scarce in the literature. Permeation of MEL across the cell membrane has been also a subject of study and debate. So, recent studies indicated that cellular permeation rates in the pineal gland are of the order of 1.7 *μ*m/s^42^ and that they can occur by pure diffusion. Conversely, some studies found that active processes are required for the entrance of melatonin inside cancer cells^43^. In particular, glucose transporters (GLUT family) may play a central role in melatonin uptake and even be able to inhibit tumor growth^44^.

Molecular dynamics (MD) simulations have been proved to be a highly successful tool for the modeling and simulation of atoms, ions and small solutes in bulk and at interfaces, sometimes under strict confinement conditions^45–47^. Regarding the interaction of water, ions and small molecules at cell membranes^48–51^, several reliable force fields such as AMBER, OPLS and, especially CHARMM^52–55^ have been developed. These works include also investigation on the role of small molecules such as melatonin in solvated phospholipid membranes^56–60^. Nevertheless, the theoretical study of molecular binding to membranes is a computationally demanding task due to the long simulation times required to probe association and dissociation events. In addition, in a system with multidimensional reaction coordinates, usually several stable states (bound configurations) are separated by high free energy barriers corresponding to transition states of the system^61,62^, making it difficult for MD simulations to sample them adequately^63^. Free energy calculations using enhanced sampling techniques provide a method to address the problem and provide a great deal of understanding of the passive diffusion phenomena of small solutes over a barrier, which requires a detailed view of the underlying free energy surface (FES). However, despite the significant progress of free energy calculations achieved in recent years^64–66^, to the best of our knowledge the free energy landscape of melatonin and most of small solutes on membrane surfaces is still mostly unknown. This is partially due to the difficulty of applying appropriate sampling techniques to address the problem, and also because of the complexity of the membrane environments, making the determination of the proper collective variables a difficult challenge^67^.

The problem of computing free energy landscapes in multidimensional quantum or classical systems has been extensively discussed in the literature. A wide variety of methods has been proposed, such as hybrid quantum mechanics/molecular mechanics methods^68^, some of them without the need of a previous knowledge of reaction coordinates such as transition path sampling^69–74^, others based on multidimensional order parameters like adaptive biasing force^75^ for instance or, most of them, on the basis of preconceived reaction coordinates, such as umbrella sampling methods^76,77^, which normally allow to place the probe at different one-dimensional coordinates (for instance, the *Z*-axis normal to the instantaneous plane of the membrane). Further, density functional theory (DFT) molecular dynamics^78^ or calculations of potentials of mean force^79^ based on reversible work methods^58^ use order parameters such as the radial distance between atomic sites. However, other degrees of freedom orthogonal to the biased one (*z*) are often of importance, and if these degrees of freedom are not properly sampled, the obtained FES will contain errors that could easily lead to wrong conclusions. In this work we have employed well-tempered metadynamics, a method able to efficiently explore free energy surfaces of complex systems using multiple reaction coordinates (instead of a single one) what has been revealed to be very successful^63^ for a wide variety of complex systems^80–84^. Furthermore, we will also discuss the role of cholesterol in the cellular absorption of small molecules.

## Methods

The calculation of Helmholtz or Gibbs free energy differences for a realistic condensed matter system is a difficult task, mainly due to the fact that the partition function of a multidimensional system is normally unknown. In the present case, our main aim is to obtain free energy differences in the process of binding/crossing of a small molecule at/through at model cell membrane. This will involve configurational changes and, consequently, it will require a considerable amount of computational time in order to explore and obtain a precise knowledge of the hypersurface of potential energy of the system^85^. This task can be done by means of a variety of methods, as we have highlighted above. All the aforementioned are suitable but their computational cost is extremely high. A method called “local elevation” (Huber *et al*.^86^) or “conformational flooding” (Grubmüller^87^) was the initial idea that lead to Laio and Parrinello^63,88^ to introduce the concept of metadynamics as a method to explore multidimensional free energy surfaces of complex systems as a function of a finite number of the so-called *collective variables* (CV) which are *a priori* unknown. CVs are arbitrarily chosen and act as effective reaction coordinates to drive the calculations when moving the probe between stable state basins surrounded by free energy barriers at the multidimensional configuration space.

Metadynamics has a clear advantage over methods like umbrella sampling: an initial estimate of the energy landscape to explore is not requiered. However, a right selection of the CVs is crucial for the sake of the efficiency of the calculation. Given some drawbacks of the original method, a new version was released. Well-tempered metadynamics^89,90^ is a variant of metadynamics able to enhance the sampling of multiple CV dimensions.

In this work we have used 1.4 *μ*s metadynamics simulations to perform Gibbs free energy calculations of the binding states of melatonin at phospholipid membrane surfaces made by DMPC lipids and cholesterol in sodium-chloride aqueous solution. We first performed three 200 ns unbiased MD simulations using the GROMACS/2018.3 package in order to minimize and equilibrate the three sets of MEL-membrane systems, before launching the well-tempered metadynamics simulations. The specific interactions of MEL with cell membranes are revealed and interpreted from a free energy perspective, providing a quantitative characterization of the barriers between stable states as well as of the most relevant binding states of MEL to water and lipids. Our work employs a general methodology to define meaningful reaction coordinates as well as to explore free energy landscapes for small molecules or drugs at complex biological interfaces which can be extended to study other interactions of interest between such species and charged head groups in colloidal chemistry and biology^91^. All technical details have been reported in the Supplementary Information (SI).

**Figure 1:**
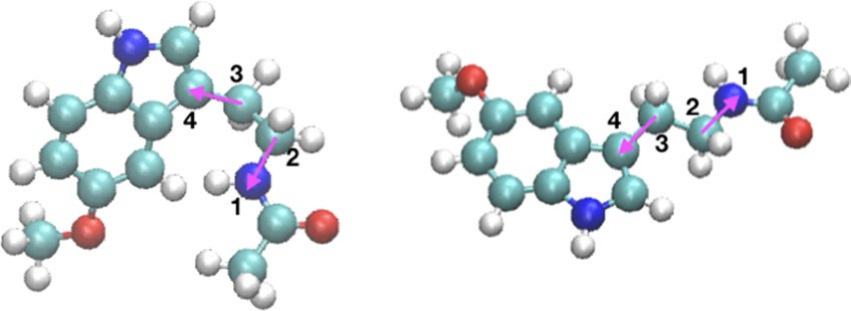
Two structural configurations of MEL from the geometrical point of view: folded (left) and extended (right). The dihedral (torsional) angle Ψ (defined by means of the sites 1-2-3-4 indicated in the figure by the two magenta arrows) taken as one of the CV in the present work. The atoms forming the melatonin molecule are: carbon (cyan), oxygen (red), hydrogen (white) and nitrogen (blue).

For membranes in ionic solutions, there exists a competition between ion binding to water molecules and to certain binding sites of phospholipids and, eventually, cholesterol^64,66,92,93^. Further, interaction between several classes of lipids and cholesterol can play a significant role as well^54^. In such a case, a bound state can be characterized by variables such as the coordination number of the ions with lipid/cholesterol binding sites and its simultaneous coordination number with water molecules^93^. In the present case, we observed from unbiased MD simulations of MEL in DMPC membranes^60^ that two orientations of MEL can be associated to three different dihedral angles involving particular molecular directions. However, only one of such dihedral angles, namely a given angle Ψ shows an angular distribution clear enough to serve as a suitable CV, since this particular angle reaches the same two stable values (1.42 ± 0.41 rad and 2.96 ± 0.18 rad) at all three cholesterol concentrations, corresponding to two preferential molecular configurations adopted by MEL, that we called “folded” and “extended”, respectively. The remaining dihedral angles evaluated showed large fluctuations and were clearly inadequate as CVs. Accordingly, we defined two main CVs to describe the binding states of MEL: CV1 is the dihedral angle Ψ, as represented in Fig. 1 and CV2 is the distance ${z}_{{\rm{MEL}}-{\rm{DMPC}}}$ between the center of mass of the melatonin molecule and the center of the DMPC bilayer membrane, defining *Z*-axis as the direction normal to the instantaneous plane of the membrane, i.e. the plane formed by axes *X* and *Y*. From here on, we will refer to CV2 simply as *z*. We should point out that in previous standard MD simulations we have observed that MEL is normally bound to cholesterol and/or to the carboxyl group of DMPC and in a less usual manner to the phosphate groups of DMPC (see ref. ^60^) and forming always a dihedral angle of ±*π* rad, i.e. at the extended configuration.

## Results and Discussion

### 2D Free energy landscapes

**Figure 2:**
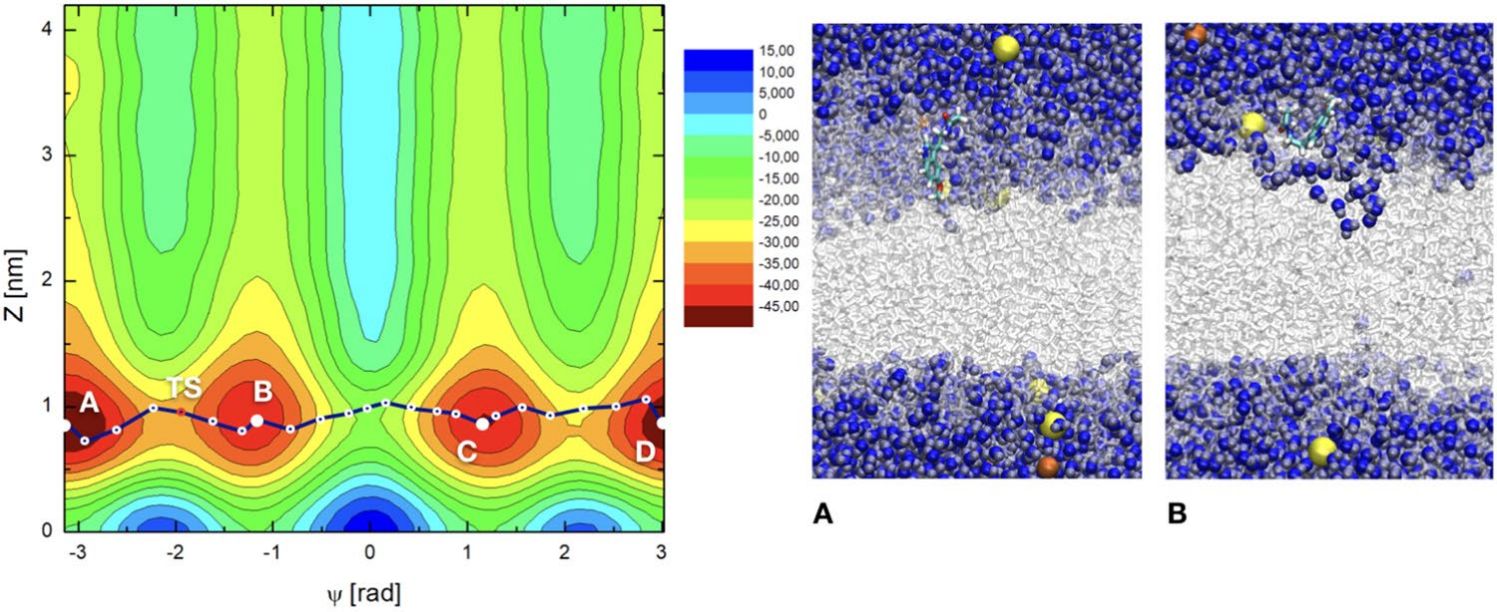
2D free energy landscapes *F*(Ψ, *z*) (in kJ/mol) in the cholesterol-free case. Four stable state basins (A,B,C,D) are indicated. TS indicates a (local) transition state between basins (**A,B**). The minimum free energy path is shown in dark blue, indicating all the computed points of the path, as listed in Table 3 of SI. Snapshots ‘A’ and ‘B’ correspond to selected basins (**A,B**) on the free energy hypersurface, respectively. In between basins (**A,B**) we have found a transition state corresponding to the conformational angular change of MEL between extended (basin A) and folded (basin B) configurations. DMPC (white), sodium ions (yellow), chlorine (dark orange), water (blue) and for melatonin: carbon (cyan), nitrogen (dark blue), oxygen (red), hydrogen (white).

**Figure 3:**
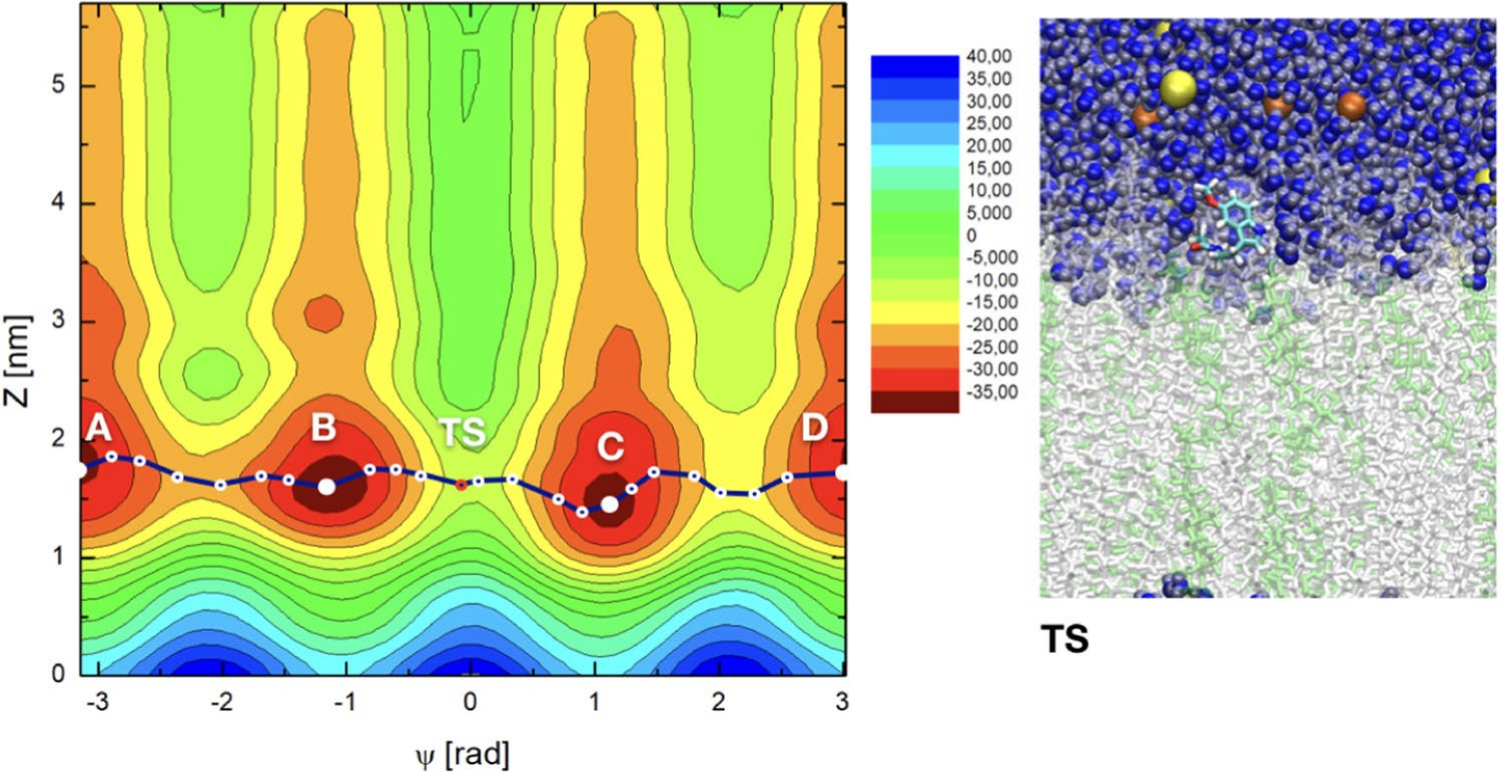
2D free energy landscapes *F*(Ψ, *z*) (in kJ/mol) in the case of 30% cholesterol. Minimum free energy path pictured as in Fig. 2. Snapshot ‘TS’ corresponds to the global TS on the free energy surface. Colors as in Fig. 2, with cholesterol chains depicted in green.

**Figure 4:**
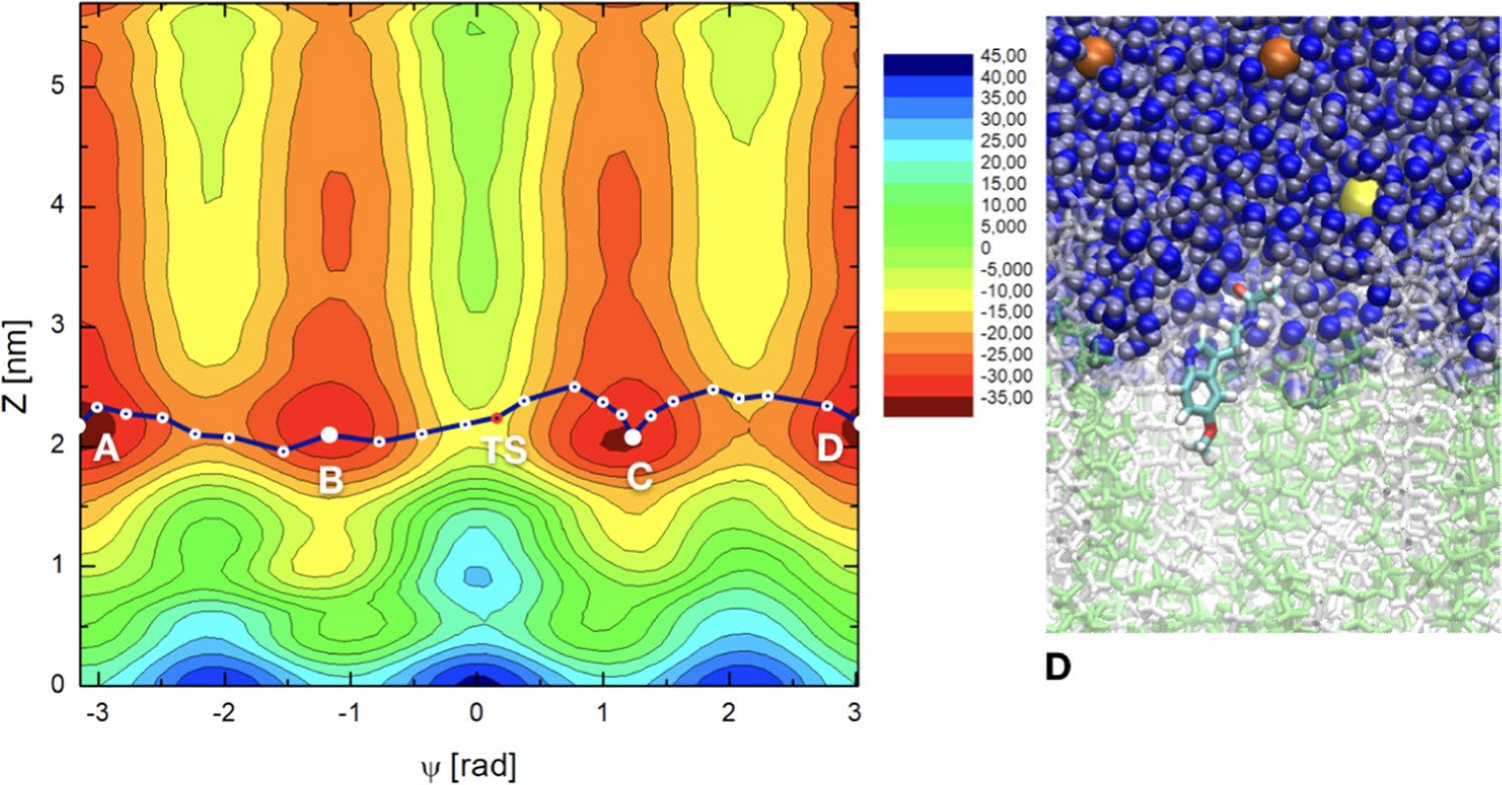
2D free energy landscapes *F*(Ψ, *z*) (in kJ/mol) in the case of 50% cholesterol. ‘TS’ corresponds to the global TS on the FES. Snapshot ‘D’ corresponds to selected basin D. Colors as in Fig. 3.

Three sets of two-dimensional (2D) well-tempered metadynamics simulations based on the specific CVs defined above were performed to calculate free-energy surfaces of MEL at cholesterol contents of 0%, 30% and 50% at neutral zwitterionic DMPC membranes. The results reported in Figs. 3–6 of SI clearly indicate that all the simulations converged properly. The resulting 2D free energy surfaces of melatonin bound to DMPC membranes are shown in Figs. 2–4. Each state can be indexed by the two CVs. A pattern including several regions with clear minima is present in the FES in all cases. The main features are the global minima of each FES located between $z\in \mathrm{[0.7,2.3]}$ nm and around two distinctive values for the dihedral angle, namely those around $|\Psi | \sim [1.2,\pi ]$ rad. Such orientations are in excellent agreement with the average values of Ψ obtained from ordinary MD simulations^60^ (see Fig. 7 therein) and they correspond to the two folded and extended geometries of melatonin previously observed.

As a general feature, free energy values from metadynamics simulations collected from the 2D-surfaces correspond to contour plots with values including a global minimum. Usually, this global minimum is set to zero. Nevertheless, we should point out that the zero of each 2D plot can be arbitrarily fixed, since the physically meaningful quantities are not the absolute values of *F*(Ψ, *z*) but the free energy differences. In the present work, the zeroes of each 2D plot have been set at the configuration of MEL fully solvated for water, since the extracellular bulk is a common feature of all sets, regardless of their cholesterol contents. In particular, we set the zero free energy for values of CV2 (*z* positions) at ∼4 nm (cholesterol free case) and ∼5 nm (for the two cholesterol-rich cases) i.e. with MEL fully solvated by water, and the values of CV1 corresponding to the lowest value of F, that was for Ψ = 0 in all cases. This means that, since we did not set the zero at the absolute minimum value of F for each 2D surface, we are reporting positive and negative values of *F*.

The overall range of absolute free energies reported in Figs. 2–4 is rather wide, similar to the range reported in the work of Jämbeck and Lyubartsev^64^ for small-molecules (ibuprofen, aspirin and diclofenac) around lipid bilayers, who found free energy ranges of the order of 70 kJ/mol and barriers of around 20–40 kJ/mol. On the other hand, in our preliminary unbiased MD simulation^60^ we estimated from reversible work calculations (based on considering radial distances as reaction coordinates) barriers for MEL forming or breaking hydrogen bonds to DMPC lipids to be in between 2 and 10 kJ/mol, when MEL is located inside the interfacial region, but free energy barriers related to orientational changes of MEL and to global displacements of MEL to the center of the membrane or to the extracellular bulk were not considered. Given this wide free energy range, we will focus especially on the characterization of the free energy barriers between the particular states of MEL at interfaces and MEL fully solvated by water, where the raw differences are in the range of 10–25 kJ/mol.

Further, in Fig. 2 of SI we can clearly identify a few events where MEL permeated the membrane from one leaflet to the other one at all three cholesterol concentrations, because of the bias employed in the well-tempered metadynamics runs and also that the two selected CVs reached all possible values within given ranges. Nevertheless, in the time span of 200 ns of auxiliary MD simulations we did not record any spontaneous crossing of MEL through the DMPC membrane. Given the open debate on the mechanisms of MEL permeation through cells (see References^43,44^), our results appear to be in qualitative disagreement with the reported experimental permeability of MEL across the pineal gland plasma membrane^42^, of the order of 1.7 *μ*m/s. We believe that the disagreement is essentially due to the pressure difference between both sides of the plasma membrane applied in the experiments by Yu *et al*.^42^ and that we did not consider in the present work where, as indicated in SI, we performed our simulations at the NPT ensemble considering a constant pressure of 1 atm at the two sides of the membrane and for the whole system. In addition, the experimental setups consider real plasma membranes containing a large variety of species such as large trans-membrane proteins that we have not considered here and that may favor the crossing of the membrane by MEL. Finally, in the recent work of Wang *et al*.^59^ small solutes such as glycerol, caffeine, iso-propanol or etho-suximide were simulated nearby a model cell membrane. These authors found that, in order to observe trans-membrane crossings of such small solutes in the time length of a simulation at the atomic level of description, they needed to run trajectories of 10 *μ* s at low temperatures (310 to 330 K) or, alternatively, rise the temperatures to more than 400 K (for simulation times of 1 *μ*s).

The 2D free energy landscapes reveal that the most favorable stable states of melatonin binding to the membrane (basins A,B,C,D) correspond to *z*-distances around 0.8 nm at the cholesterol-free system, whereas such distance tends to increase significantly around to 1.6 nm for the 30% cholesterol concentration and up to 2.2 nm when cholesterol reaches 50% of the total amount of lipids in the system. Considering the information revealed by CV1, i.e. the torsional angle Ψ, we can distinguish two sets of minima: (1) For |Ψ| ∼ 1.2 rad (basins B and C) and (2) for |Ψ| = *π* rad (basins A, and D). These minima are related with the two preferential configurations of MEL close to a DMPC-cholesterol bilayer (folded, extended) indicated above. According to CV2, i.e. the distance between the center of mass of the melatonin molecule and the center of the DMPC bilayer membrane, MEL is preferentially located at the interface of the DMPC-cholesterol bilayer (regions with 0.7 < *z* < 3.0 nm).

Locations of MEL outside the interface i.e. (*z* < 0.7 and *z* > 3.0 nm) show large free energies. These regions will be considered as (1) “water-solvated” (*z* > 3.0 nm, assuming that it in such regions MEL is fully solvated by the electrolyte solution surrounding the membrane) or (2) “internal” regions of the membrane (*z* < 0.7 nm, assuming that in such cases MEL is fully imbedded into the body of the membrane). In previous works, related to the structure and dynamics of tryptophan^41,94^ we observed that tryptophan, a molecule quite similar to melatonin (and one of its precursors) spent about 30% of unbiased MD simulation runs at the “water-solvated” state and the remaining time adsorbed at the interface. This state can be considered as a normal, accessible configuration of MEL. As it can be observed in Figs. 2–4 when MEL is fully solvated by water (4.2 > *z* > 3 nm for the cholesterol-free case and 5.7 > *z* > 3 nm at 30 and 50% cholesterol) it tends to stay in a quite high free-energy conformation, far away from the corresponding minima, located at the stable state basins. In this situation MEL can change its orientational order with a moderately low free energy cost. Something similar happens when MEL reaches very internal regions of the membrane (*z* < 0.7 nm).

Since the present 2D representation includes a wide variety of low free energy states related to conformational and structural changes, some specific numerical calculations are in order. In particular, it is very useful to compute the so-called *minimum free energy path* (MFEP), that can be determined by iteratively refine a pathway connecting stable states that converges to the minimum free energy trajectory between them (see References^95–98^). Although no full explanation of these methods is reported here, particular details about how minimum free energy paths have been obtained are reported in SI. There we have included Table 3, where the coordinates of the three paths are listed.

From MFEP we can extract information associated to the most probable trajectories in the CV space followed by the system when evolving between stable states and when eventually crossing high energy barriers that can be associated to local or global transition states (TS) located at the free energy hypersurface. Here we assume the canonical interpretation give by Transition State Theory of the (global) TS as a saddle point in between two stable states located at the minimum free energy path and having the largest energy^99,100^. In the following list we will report the size of free energy barriers at two given states. These barriers can be compared to experimental data (see for instance ref. ^98^). The main features and numerical estimations can be summarized as follows:

1. Measuring the value of the absolute free energy directly from the 2D plots, we can identify the approximated location of TS revealed by the MFEP:

- In Fig. 2, the local maximum labeled TS is at the pair of coordinates (Ψ = −1.97 rad, *z* = 0.97 nm);
- The global TS in Fig. 3 is located at (Ψ = −0.14 rad, *z* = 1.58 nm) and
- The global TS in Fig. 4 corresponds to (Ψ = 0.16 rad, *z* = 2.25 nm).

2. We can estimate the location and values of the free energy barriers between the significant states of “MEL at interface” and “water-solvated” (taken in all cases at *z* = 4 nm) respectively for each cholesterol concentration. Let us note coordinates as (CV1 in rad, CV2 in nm):

- At 0% cholesterol, MEL at interface corresponds to the coordinate (−*π*, 0.79) and when water-solvated corresponds to (−*π*, 4.0), with a barrier is of 25.3 kJ/mol;
- At 30% cholesterol, the two coordinates are (−1.19, 1.41) and (−1.19, 4.0) with a barrier of 14.1 kJ/mol;
- At 50% cholesterol, the two coordinates are (−*π*, 2.17) and (−*π*, 4.0) with a barrier of 9.1 kJ/mol.

3. Other relevant free energy barriers are located between the free energy minima (let us take basin ‘A’ as the reference) and the center of the membrane (*z* = 0) in all cases, with values of the order of 40 kJ/mol (0% cholesterol) and of 50 kJ/mol (for 30% and 50% cholesterol). These barriers are difficult to be crossed spontaneously by MEL, but they are interesting in order to estimate the amount of free energy such that MEL can permeate the membrane. As expected, when cholesterol is present in the membrane, the barriers are considerably higher than for the cholesterol free case.
4. Concerning angular-related barriers, there is a relevant one between the two angular stable states of MEL (basins A and B), i.e. folded (|Ψ| ∼ 1.17 rad) and extended (|Ψ| = *π*) conformations of 18.8 kJ/mol for the cholesterol-free case and of 19.7 kJ/mol at 30% and 17.6 kJ/mol at 50% cholesterol concentrations. This corresponds to MEL at the interface. This conformational barrier corresponds to surmount the local TS indicated in Fig. 2. The corresponding barrier when MEL is solvated by water is of 18.3 kJ/mol.
5. Other TS can be obtained for the conformational change between angles Ψ = −1.17 rad and Ψ = 1.17 rad (i.e. between basins B and C in Figs. 3 and 4). The corresponding barriers are of 22.0 kJ/mol (30% cholesterol) and 28.3 kJ/mol (50% cholesterol). These can be considered the absolute TS of the system, since they show the maximum free energy along the reaction coordinate, indicated by the MFEP. The equivalent global TS of Fig. 2 (0% cholesterol), has a barrier of 26.5 kJ/mol. In all cases, MEL crossing of a TS can be carried out through an intermediate configuration of MEL with Ψ = 0, as represented in the snapshot reported in Fig. 3. When MEL is solvated by water, the corresponding barrier is of about 21 kJ/mol for each case.

The visible shifts to larger distances *z* of basins A, B, C and D shown in Figs. 2–4 clearly reveal that the binding competitiveness of lipid head groups has been diminished by cholesterol contents and that the affinity of MEL to DMPC membranes becomes less favorable as cholesterol concentration increases, which is in agreement with previous experiments and simulations on MEL and cholesterol at variable concentrations close to dioleoylphosphatidylcholine and dipalmytoilphosphatidylcholine membranes^56,57^, where it was observed that cholesterol helps a membrane to increase its thickness and reduce its area per lipid, whereas melatonin operates in the reverse way and tends to decrease the thickness of the membrane and increase its area per lipid.

**Figure 5:**
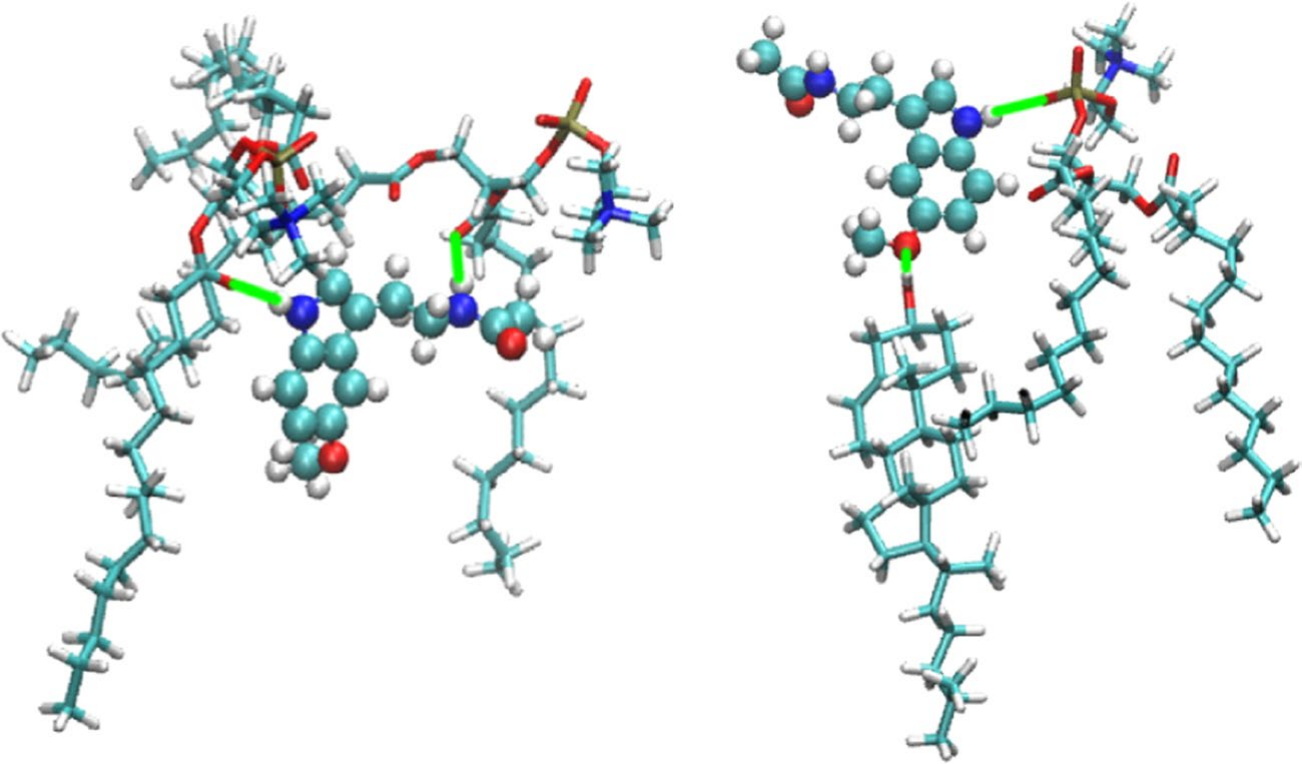
Snapshots of representative bound states for MEL at the interface of the membrane. Colors of MEL as in Fig. 1. Colors for DMPC/cholesterol: carbon (cyan), nitrogen (dark blue), oxygen (red), hydrogen (white). Hydrogen bonds indicated as green solid lines. Left: case of 0% cholesterol concentration, with MEL bound to two DMPC molecules; Right: case of 30% cholesterol concentration, with MEL bound to one DMPC and one cholesterol molecule.

In order to show characteristic molecular configurations of MEL when located at the interface of the membrane, two characteristic snapshots at 0 and 30% cholesterol concentrations are reported in Fig. 5. We have included in the figure only MEL and its closest solvating lipids, namely two DMPC at 0% and one DMPC and one cholesterol at 30%. Both configurations correspond to the dihedral angle Ψ = *π* rad and to *z* distances of 0.8 (0%) and 1.6 nm (30%). With the aid of these snapshots we can see that MEL is able to be simultaneously bound to both DMPC and cholesterol chains through hydrogen-bonds. The binding sites were determined to be oxygens ‘O6’ of DMPC and the oxygen from the hydroxyl group of cholesterol and hydrogens ‘H15’ and ‘H16’ of MEL (see Reference^60^ for further details).

According to point (1) of the list above, the energetic cost for MEL to move from the interface to the water-solvated region diminishes when cholesterol concentration increases. This means that cholesterol favors MEL to move outside the membrane and eventually stay in the outer regions of the interface. To understand these changes, we should remember the well-known condensing effect of cholesterol on lipid bilayers, which produces higher membrane rigidity and ordering^101^. Interestingly, such transition in the binding behavior of MEL at cholesterol ≈30% mol might be correlated with the phase transition point of DMPC-cholesterol membranes, in which membranes change from a liquid-disordered phase to a liquid-ordered phase^102,103^. Concerning the angular configurations, our results indicate that changing from extended to folded forms does not require to surmount a high energy barrier, but one of about 19 kJ/mol when MEL is located at the interface of the membrane. Gibbs free energy barriers of about 15 kJ/mol have been also obtained in simulations of the translocation of ethanol across a lipid bilayer membrane^59^.

### 1D free energy profiles

**Figure 6:**
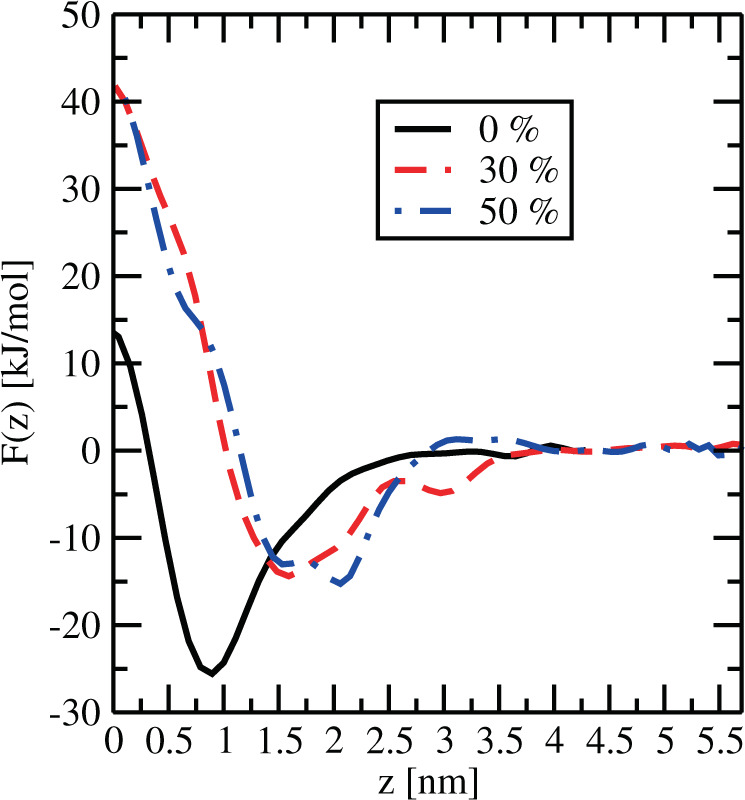
Integrated free energy F(z) for 0%, 30% and 50% cholesterol.

**Figure 7:**
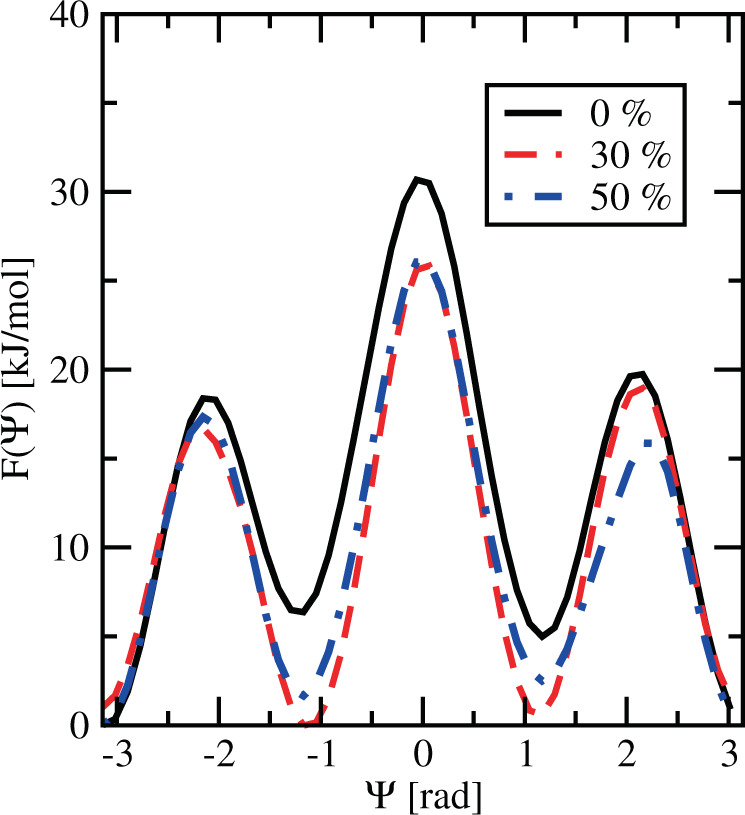
Integrated free energy F (Ψ) for 0%, 30% and 50% cholesterol.

As an alternative to the 2D FES and MFEP reported above, we considered the calculation of 1D free energy profiles as a function of only one of the two CV. This will allow us to directly compute free energy differences for meaningful MEL conformations, especially for MEL at the interface and at the center of the membrane. In Figs. 6 and 7 we represent the dependence of the integrated binding free energy *F*(*s*1) on one CV for membranes with three cholesterol concentrations after integrating out the second CV according to^64,104^:

1 $$F({s}_{1})=-\,{\beta }^{-1}\,\mathrm{ln}\,\left[\frac{\int {e}^{-\beta F({s}_{1},{s}_{2})}\,d{s}_{2}}{\int {e}^{-\beta F({s}_{1},{s}_{2})}\,d{s}_{1}d{s}_{2}}\right],$$

where *s*_1_ and *s*_2_ are the CVs, *β* = 1/(*k*_B_*T*), *k*_B_ is the Boltzmann constant and T is the absolute temperature. This means that all possible paths for the CV labeled as *s*_2_ have been averaged. *F*(*s*_1_) reveals additional information about the most stable states only as a function of one CV. This also allow us to compare the relative stability of MEL at setups with different cholesterol contents separately as a function of *z* or Ψ. These barriers can be also directly compared to experimental findings^64^.

In Fig. 6, we set the zero of each free energy at the extracellular bulk (*z* = 4 nm at 0% cholesterol and at *z* = 5 nm at 30 and 50%), as we did in the 2D free energy surfaces. Since in Fig. 7 the spatial dependence has been integrated out, we have fixed the zero to the minimum free energy in each case.

In agreement with the preliminary findings reported in the previous section, the observed behavior is that the position of stables states of MEL depends of the contents of cholesterol in the membrane: minima of *F*(*z*) are located at *z* = 0.8 nm for 0% cholesterol, at *z* = 1.6 nm for 30% cholesterol and at *z* = 2.2 nm for 50% cholesterol.

From plots in Figs. 6 and 7 we can obtain meaningful free energy barriers, i.e. free energy differences related to particular processes. We can highlight the following:

1. MEL needs about 25.5 kJ/mol to move from inside the interface of the membrane to the water-solvent regions at 0% cholesterol concentration, whereas it requires 14.3 kJ/mol at 30% cholesterol and 16.7 kJ/mol at 50% cholesterol (Fig. 6). The net effect of cholesterol is to reduce the energetic cost of MEL moving across the membrane from the interface to the aqueous solvent.
2. The barrier that needs to be surmounted for MEL to move from the interface to the center of the membrane is significantly high, between 40 (0%) and 50 kJ/mol (30–50%) (see Fig. 6). Even though these large numbers can be a drawback of the method employed here, it is very clear that in the process of crossing the membrane from one leaflet to the other, cholesterol enhances the free energy cost. We expect that increasing temperature^59^ or pressure^42^ it would be possible to observe the crossing of such barriers by MEL.
3. Further, in order to reach a folded configuration (|Ψ| = 1.17 rad) from the extended configuration (|Ψ| = *π* rad) (Fig. 7), MEL has to surmount barriers of 18.4 (0%), 15.7 (30%) and 17.3 kJ/mol (50%). This means that for MEL it is quite easy to adjust its molecular geometry in order to exchange its configuration between two orientational stable states. We should add that in standard molecular dynamics^60^, it is easy to observe that MEL can change its configuration varying the dihedral angle Ψ and that it can move from interfaces of the DMPC-cholesterol membrane bilayer to the water-solvated region and vice-versa.
4. Finally, the access to the conformational structure with Ψ = 0 demands to surmount a barrier of about 25 kJ/mol from a folded configuration. We should point out that this is essentially the cost of crossing a global TS, because in such a case the coordinate *z* changes very little (see point (5) of the list reported in “2D Free Energy Landscapes”).

In processes such as interactions of MEL with free radicals^105^, it has been estimated from DFT methods that Gibbs free energy changes from vacuum to aqueous solution were in between 33 and 670 kJ/mol. For the mechanism of MEL nitrosation (reaction of MEL with nitric oxide)^106^ the Hartree-Fock estimated free energies were of 50–59 kJ/mol *in vacuo*. Further, the Gibbs free energy of MEL binding to the protein calmodulin has been estimated to be around 36 kJ/mol, 3-fold larger than the experimental value^107^. From the experimental side, Florio *et al*.^108^ used a variety of techniques such as combination of two-color resonant two-photon ionization, laser-induced fluorescence excitation, resonant ion-dip infrared spectroscopy, fluorescence-dip infrared spectroscopy, and UV-UV hole-burning spectroscopy, to explore the conformational preferences of an isolated melatonin molecule under molecular beams. In such a system, these authors found MEL three *trans* and two *cis* conformers showing a free energy gap of about 12.5 kJ/mol, quite close to the values obtained in the present work for the conformational angular changes (folded to extended and vice-versa). Finally, Florio and Zwier^109^, analyzed the solvation of MEL by water clusters using infrared and ultraviolet spectroscopy and found barriers of about 63 kJ/mol for the *cis/trans* isomerization process. All these findings indicate the difficulty of obtaining precise values of free energy barriers in general and for processes where MEL is involved, in particular. Thus, some of these numerical estimations report values for different binding and conformational processes of order of magnitude similar to the results presented in this work.

## Conclusions

In this manuscript we have provided, to the best of our knowledge, the first quantitative characterization to date of the binding states of melatonin at model DMPC-cholesterol phospholipid cell membranes through the calculation of free energy landscapes. With the help of well-tempered metadynamics simulations we have calculated 2D free energy landscapes and located the binding stable states and several (local and global) transition states of the system. Zero of all free energies were set at the extracellular bulk, i.e. for MEL fully solvated by water and the ionic solution. Two CVs have been considered: CV1 is the dihedral angle Ψ as described in Fig. 1 and CV2 is the distance in the normal direction ${z}_{{\rm{MEL}}-{\rm{DMPC}}}$ between center of mass of MEL and of the center of lipid bilayer (given by *z* = 0). The convergence of the simulations has been found to be very good, as explained in SI. We should note that some absolute free energies reported in Figs. 2–4 are significantly high, suggesting possible drawbacks of the metadynamics method, which we did not attempted to correct.

Our results indicate that melatonin can be bound to the internal side of the membrane, at distances *z* ∼ 1–2 nm and in several stable state configurations where the dihedral |Ψ| = 1.17 rad and |Ψ| = *π* rad. Such angular values respectively correspond to structural organizations of MEL (folded and extended) with the group of atomic sites of MEL out of the indole region (that we could call the “tail” of melatonin) distributed in either closed or open (linear-like) fashion, respectively. Interestingly, the two relevant orientational configurations of MEL can be found at the full range of *z* distances to the center of the membrane, i.e., neither of them is characteristic of a particular binding either to DMPC either to cholesterol or of its solvation by water. From the calculation of the minimum free energy paths, we have observed that the most probable trajectory of MEL is along the interface of the membrane, changing its conformation between extended and folded configurations by means of surmounting free energy barriers of about 15–20 kJ/mol. In addition, the methodology employed in the present work allowed us to locate several global and local TS of the system and to estimate the values of their free energy barriers.

The role of cholesterol has been found to pull MEL off the internal regions of the interface of the membrane, i.e. to move it outside the membrane, an effect more marked as concentration of cholesterol rises. The energetic cost for MEL to leave the interface of the membrane and to be fully solvated by water (*z*-distances around 4 nm), has been estimated to be of between 10 and 25 kJ/mol. The less common situation was found to be with MEL accessing regions around the center of the membrane, process requiring to cross free energy barriers above 40 kJ/mol. We believe that the findings presented in this work could be of practical use in designing new reaction coordinates (such as a variety of torsional angles of MEL) for multidimensional, more accurate free energy calculations able to explore a wide variety of relevant small solute species of biochemical interest such as aminoacids, neurotransmitters, drugs or hormones. The same methodology can be applied to systems of larger size such as big peptides or proteins: for instance, a detailed study of the absorption and binding of the oncogenic protein KRAS-4B in dioleoylphosphatidylcholine/dioleoylphosphatidylserine/cholesterol membranes is currently under investigation in our laboratory.

## Supplementary information

Supplementary Figures

Supplementary Tables
