# Supplementary material for: Cellular absorption of small molecules: free energy landscapes of melatonin binding at phospholipid membranes
Source: Sci Rep. 2020 Jun 8;10:9235. doi: 10.1038/s41598-020-65753-z (PMC7280225; doi:10.1038/s41598-020-65753-z)
Supplement: Supplementary file 1 — Supplementary Figures [file 41598_2020_65753_MOESM1_ESM.pdf]

Supplementary information for: Cellular absorption of small molecules: free energy landscapes of melatonin binding at phospholipid membranes

Huixia Lu[1,+] **and Jordi Martí**[1,*,+]

[1]Department of Physics, Technical University of Catalonia-Barcelona Tech, B4-B5 UPC Northern Campus, Barcelona, Catalonia, Spain
[*]jordi.marti@upc.edu
[+]these authors contributed equally to this work

ABSTRACT

Figures and figure captions

1 Figures and figure captions

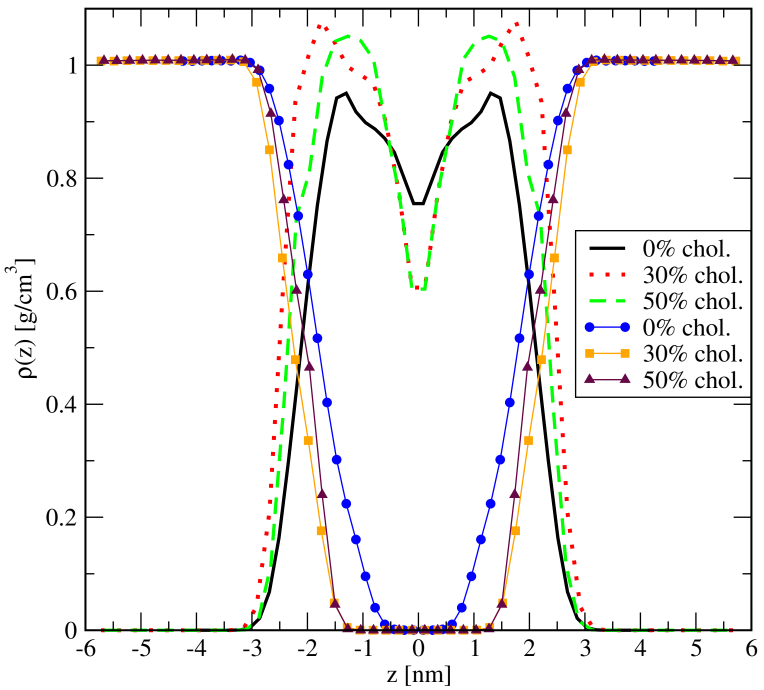

Figure 1. Density profiles of lipid backbone (full lines: 0% cholesterol, dotted lines: 30% cholesterol, dashed lines: 50% cholesterol) and water (circles: 0% cholesterol, squares: 30% cholesterol, triangles: 50% cholesterol)

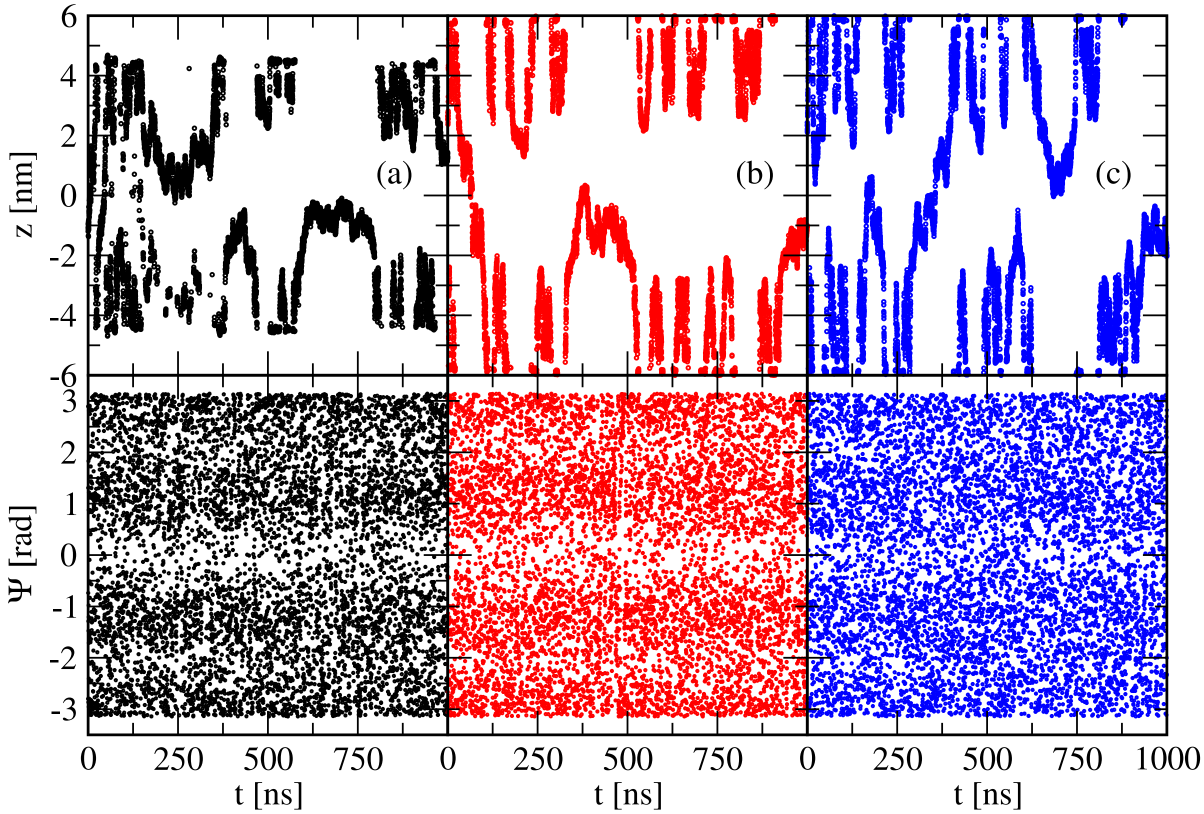

Figure 2. Fluctuations of the CV values as a function of time in different states: (a) 0% cholesterol (black), (b) 30% (red) and (c) 50% (blue).

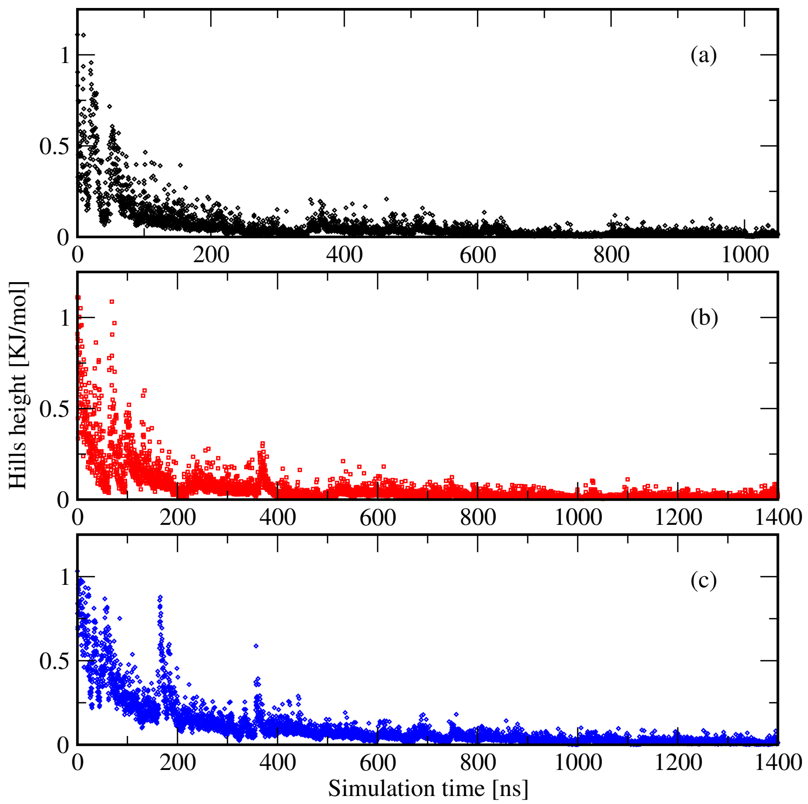

Figure 3. Well-tempered Metadynamics hills height as a function of time in different states: (a) 0% cholesterol, (b) 30% and (c) 50%.

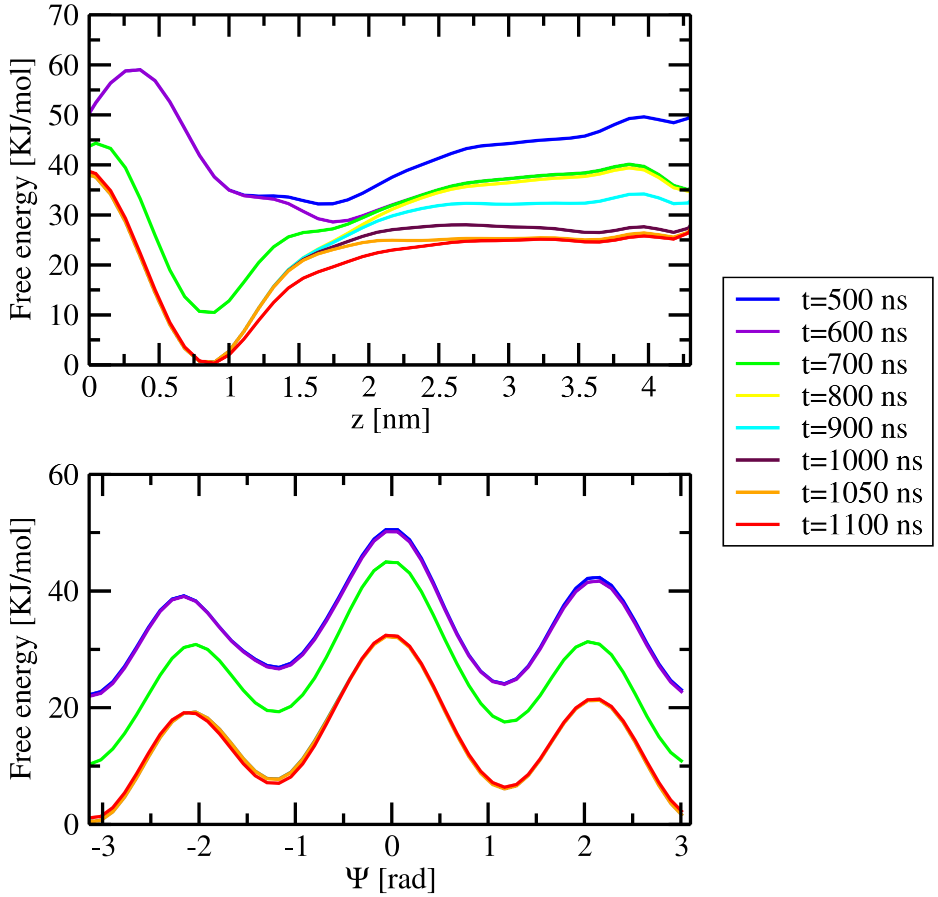

Figure 4. Time cumulative free energy profiles at the cholesterol-free system. Bottom: CV1, top: CV2.

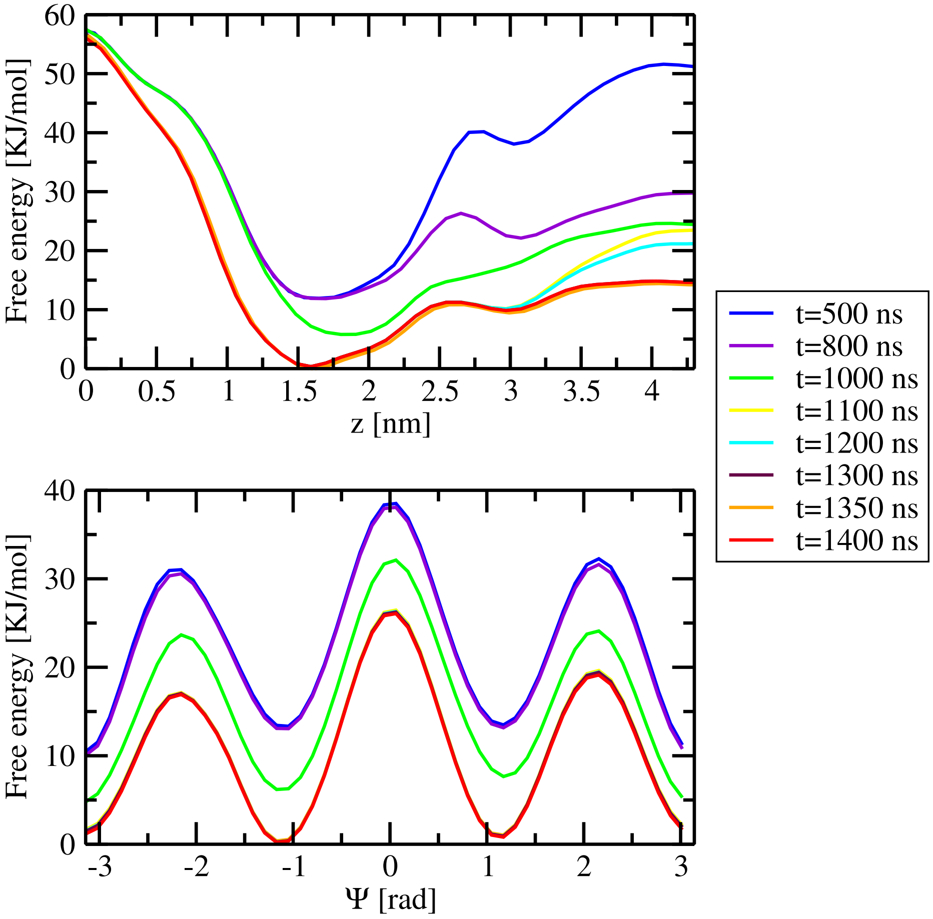

Figure 5. Time cumulative free energy profiles at the 30 % cholesterol system. Bottom: CV1, top: CV2.

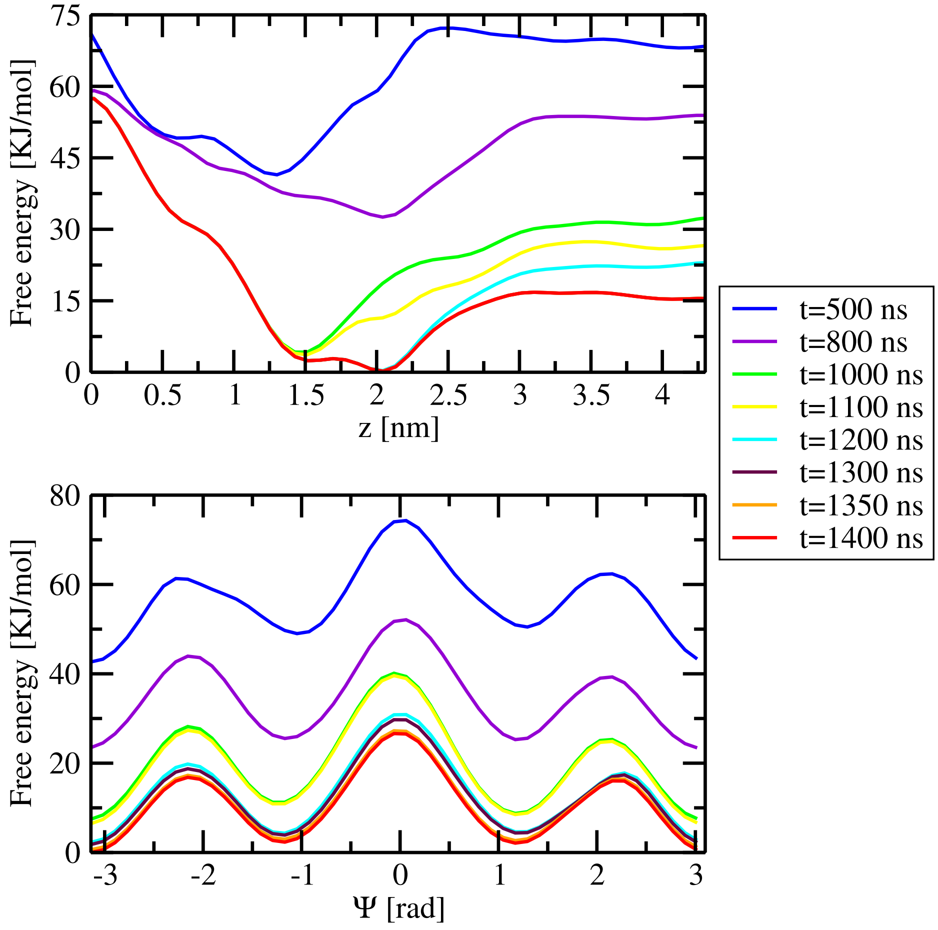

Figure 6. Time cumulative free energy profiles at the 50 % cholesterol system. Bottom: CV1, top: CV2.
