# Supplementary material for: Cellular absorption of small molecules: free energy landscapes of melatonin binding at phospholipid membranes
Source: Sci Rep. 2020 Jun 8;10:9235. doi: 10.1038/s41598-020-65753-z (PMC7280225; doi:10.1038/s41598-020-65753-z)
Supplement: Supplementary file 2 — Supplementary Tables [file 41598_2020_65753_MOESM2_ESM.pdf]

Supplementary information for: Cellular absorption of small molecules: free energy landscapes of melatonin binding at phospholipid membranes

Huixia Lu[1,+] **and Jordi Martí**[1,*,+]

[1]Department of Physics, Technical University of Catalonia-Barcelona Tech, B4-B5 UPC Northern Campus, Barcelona, Catalonia, Spain
[*]jordi.marti@upc.edu
[+]these authors contributed equally to this work

ABSTRACT

Tables

Table 1. Area per lipid and thickness of the membrane. Estimated errors in parenthesis.

Percentage of cholesterol	A (nm^2)	Δz (nm)
0%	0.618(0.012)	3.49(0.06)
30%	0.421(0.005)	4.43(0.03)
50%	0.402(0.003)	4.47(0.03)

Table 2. Simulation parameters

Parameter	0%	30%	50%
Gaussian width of CV1 [rad]	0.35	0.35	0.35
Gaussian width of CV2 [nm]	0.30	0.30	0.25
Starting (Gaussian) hill [kJ/mol]	1.0	1.0	1.0
Deposition stride [ps]	1	1	1
Bias factor	10	10	20
Simulation time [ns]	1100	1400	1400

Table 3. Coordinates of segments forming each minimum free energy path.

	Minimum free energy paths					
Stable states	0% cholesterol		30% cholesterol		50% cholesterol	
	Ψ (rad)	z (nm)	Ψ (rad)	z (nm)	Ψ (rad)	z (nm)
A	-3.14	0.79	-3.14	1.64	-3.10	2.17
	-2.96	0.66	-2.89	1.80	-2.91	2.29
	-2.64	0.77	-2.70	1.78	-2.75	2.24
	-2.26	0.99	-2.37	1.64	-2.48	2.22
	-1.97	0.97	-2.04	1.62	-2.25	2.08
	-1.58	0.88	-1.62	1.64	-1.98	2.05
	-1.32	0.73	-1.45	1.63	-1.45	1.98
B	-1.17	0.88	-1.19	1.57	-1.18	2.11
	-0.87	0.84	-0.81	1.66	-0.83	2.07
	-0.59	0.89	-0.64	1.65	-0.49	2.12
	-0.29	0.95	-0.43	1.63	-0.15	2.19
	-0.09	0.97	-0.14	1.58	0.16	2.25
	0.16	1.08	0.07	1.59	0.42	2.39
	0.44	1.02	0.33	1.60	0.70	2.47
	0.74	0.95	0.66	1.39	0.96	2.41
	0.92	0.93	0.86	1.36	1.08	2.26
C	1.17	0.84	1.12	1.51	1.20	2.02
	1.29	0.90	1.32	1.57	1.37	2.24
	1.58	0.98	1.49	1.70	1.50	2.33
	1.80	0.97	1.76	1.56	1.80	2.45
	2.30	0.99	2.02	1.50	2.05	2.35
	2.59	1.00	2.31	1.41	2.48	2.37
	2.87	1.06	2.52	1.62	2.73	2.26
D	3.03	0.80	3.03	1.69	3.05	2.19
